# Endovascular repair of traumatic innominate artery injury: case report

**DOI:** 10.1093/jscr/rjag539

**Published:** 2026-06-29

**Authors:** Fajer Al-Ishaq, Hassan Al-Thani, Ayman El-Menyar, Ahmed Sadek, Ahmad Zitoun, Anas Aldakhl-Allah, Ahmed Hussein

**Affiliations:** Department of Surgery, Hamad Medical Corporation, PO Box 3050, Doha, Qatar; Department of Surgery, Vascular Surgery, Hamad Medical Corporation, PO Box 3050, Doha, Qatar; Clinical Research Trauma and Vascular Surgery, Hamad Medical Corporation, PO Box 3050, Doha, Qatar; Clinical Medicine, Weill Cornell Medicine, PO Box 24144, Doha, Qatar; Department of Surgery, Vascular Surgery, Hamad Medical Corporation, PO Box 3050, Doha, Qatar; Department of Surgery, Vascular Surgery, Hamad Medical Corporation, PO Box 3050, Doha, Qatar; Department of Surgery, Vascular Surgery, Hamad Medical Corporation, PO Box 3050, Doha, Qatar; Department of Surgery, Vascular Surgery, Hamad Medical Corporation, PO Box 3050, Doha, Qatar

**Keywords:** trauma, innominate artery, brachiocephalic, endovascular stenting, vascular injury

## Abstract

Traumatic injury to the innominate artery (IA) is an uncommon vascular condition with a prehospital mortality up to 70%. It may result from high-speed blunt chest trauma. We present a 22-year-old male motorcyclist who was struck by a car. The patient sustained multiple injuries and the initial assessment revealed a hemopericardium and a widened mediastinum. Following stabilization, whole-body imaging showed an intimal flap of the IA with contained contrast extravasation, and a lung laceration. The patient underwent endovascular stent grafting of the IA pseudoaneurysm via femoral artery access. Subsequently, he required maxillofacial and orthopedic procedures. At the time of rehabilitation, he was ambulating independently with minimal assistance and expressed satisfaction with his return to routine. Blunt IA injury is a rare but life-threatening vascular trauma that necessitates rapid diagnosis and tailored management. This case highlights that endovascular stent grafting may serve as a safe, minimally invasive alternative to conventional open surgical repair in appropriately selected patients.

## Introduction

Traumatic innominate artery injury (IAI) carries devastating consequences, with > 70% of patients dying before reaching the hospital [1]. The innominate artery (IA), also called the brachiocephalic trunk, is a major blood vessel arising from the aortic arch that supplies blood to the right arm, head, and neck. Although the IA is shielded by the rib cage, IAIs are rare but can be fatal [2]. These injuries may result from direct trauma or from other thoracic injuries, such as fractures of nearby bones like the clavicle, sternum, or ribs. Trauma to the IA can result from penetrating injuries (such as stab wounds), blunt force (such as vehicle accidents), or medical procedures (iatrogenic injuries), all of which may damage the artery’s wall [3].

Early diagnosis and management of IAIs are critical. Failure to identify and treat these injuries often leads to catastrophic outcomes. Advanced imaging modalities are pivotal for evaluating trauma patients with suspected vascular injuries. Management (open surgical or endovascular repair) varies by patient condition and hospital experience [1]. We present a case of a young male with blunt IAI, among other injuries, who was treated with endovascular stenting.

## Case report

A 22-year-old male motorcyclist, wearing a full-face helmet, was struck by a car. He sustained multiple injuries and was taken to a Level 1 trauma center. The emergency medical services found him unstable with a Glasgow Coma Score of 6. He was intubated at the scene and started on vasopressor support. In the trauma room, his heart rate was 156 beats/min, blood pressure was 103/68 mmHg, and mean arterial pressure (MAP) was 82 mmHg. He received mechanical ventilation with FiO_2_ at 100% and oxygen at 15 L/min. Vasopressor infusion was titrated to optimize the MAP before intervention.

**Figure 1:**
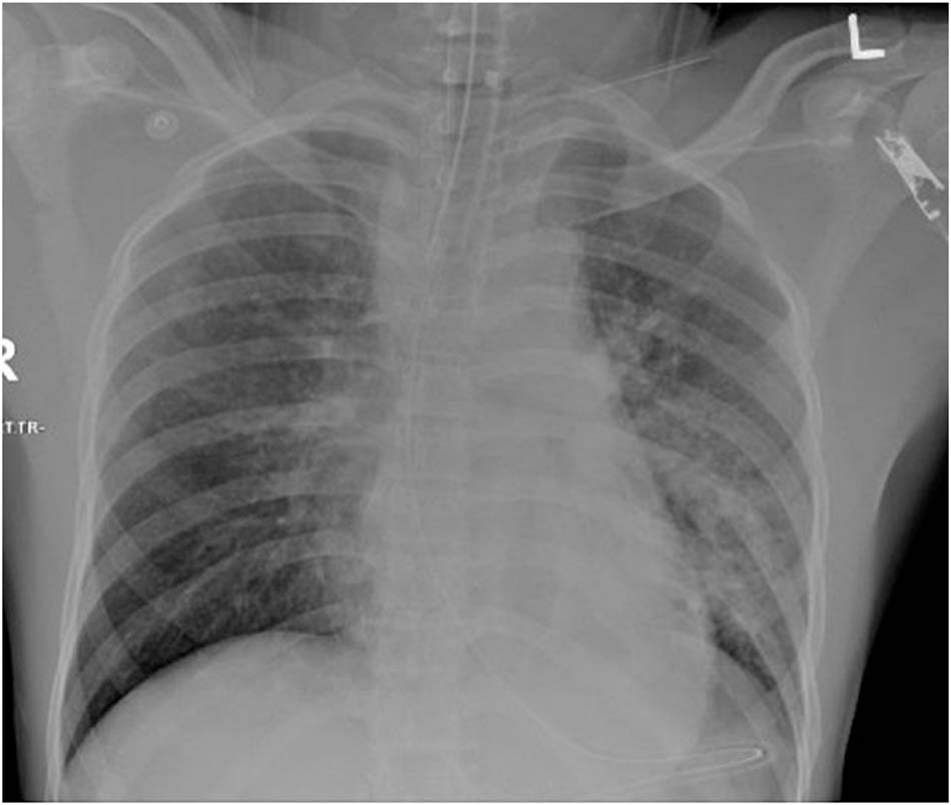
Initial chest X-ray showing widen mediastinum.

**Figure 2:**
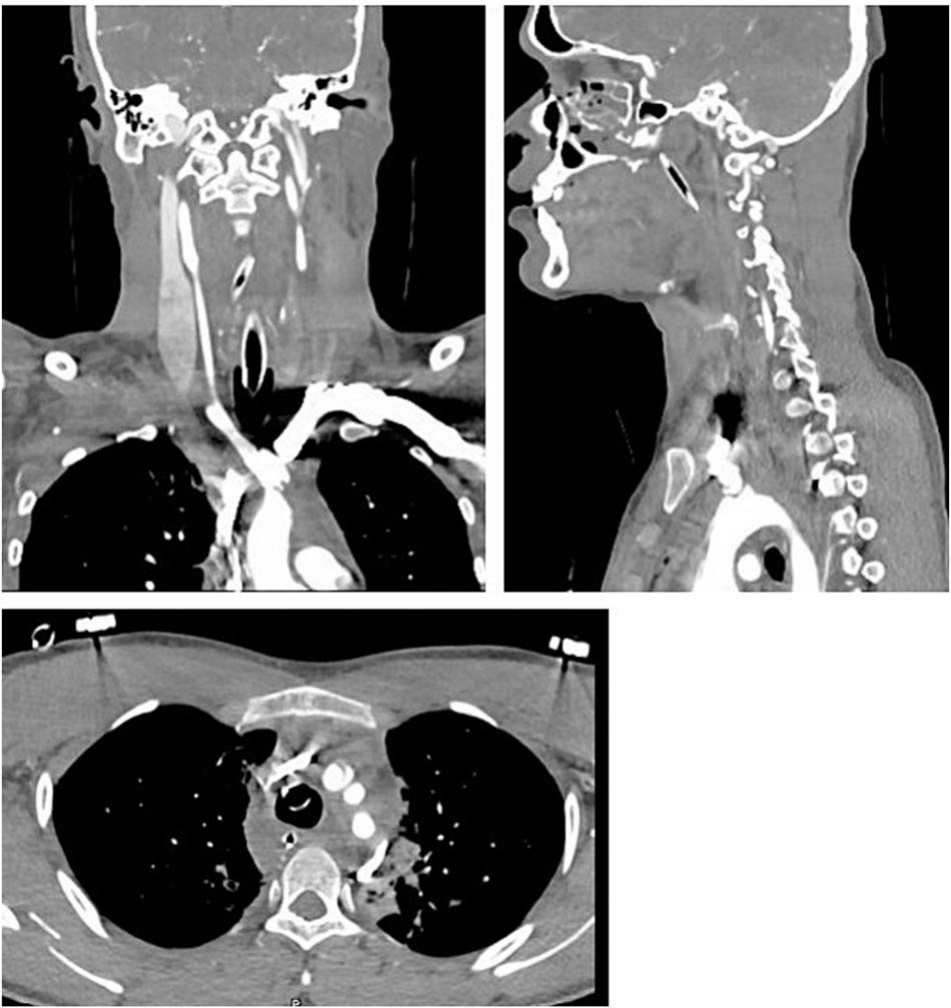
Initial CT scan showing IA flap with tiny extravasation of the contrast.

Extended focused assessment with sonography in trauma showed hemopericardium, and chest X-ray revealed a widened mediastinum (Fig. 1). The whole-body computerized tomography scan revealed a hemopericardium, an IA intimal flap with contained contrast extravasation (Fig. 2), a left lung laceration, alongside mild intracranial hemorrhages, a mild pericardial effusion, facial fractures, and skeletal injuries. The intimal flap, measured ~ 9 mm, was located 5 mm from the IA origin and did not involve the origin of the carotid or subclavian branches, which was confirmed by computed tomography (CT) angiography. This favorable flap length and branch proximity supported the decision to defer open repair in favor of an endovascular approach.

**Figure 3:**
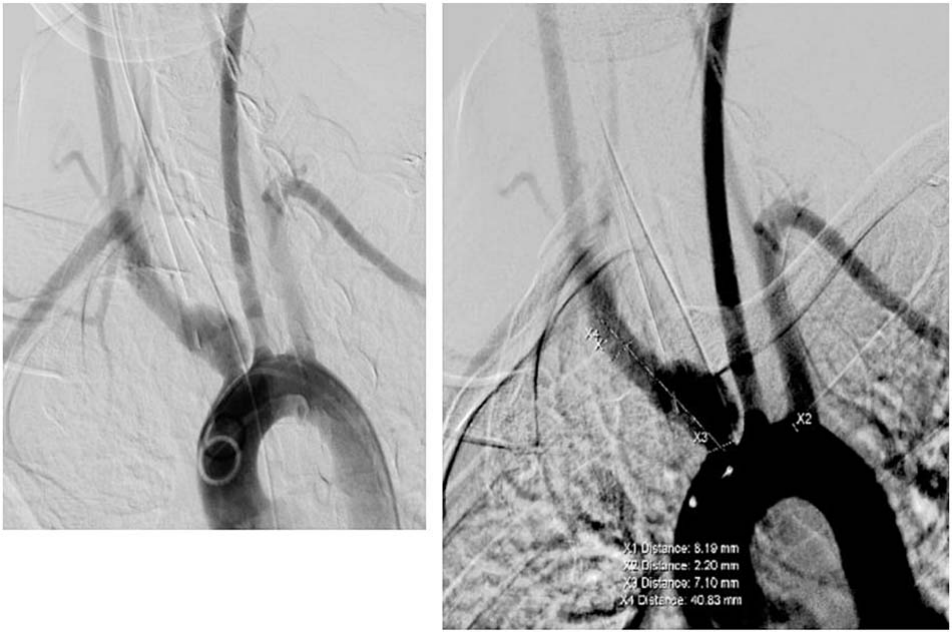
Angiogram showing IA aneurysm.

Vascular and cardiothoracic surgery teams recommended a follow-up CT in 6 h. He was admitted to the trauma intensive care unit for stabilization. On hospital Day 5, a CT angiogram was limited by artifact but confirmed the presence of an IA intimal flap. On hospital Day 10, the team performed an aortic arch aortogram in a hybrid operating theater. The aortogram revealed a pseudoaneurysm (18 × 13 mm) located 5 mm from the IA origin, with proximal and distal diameters of 7 and 8 mm, respectively (Fig. 3).

**Figure 4:**
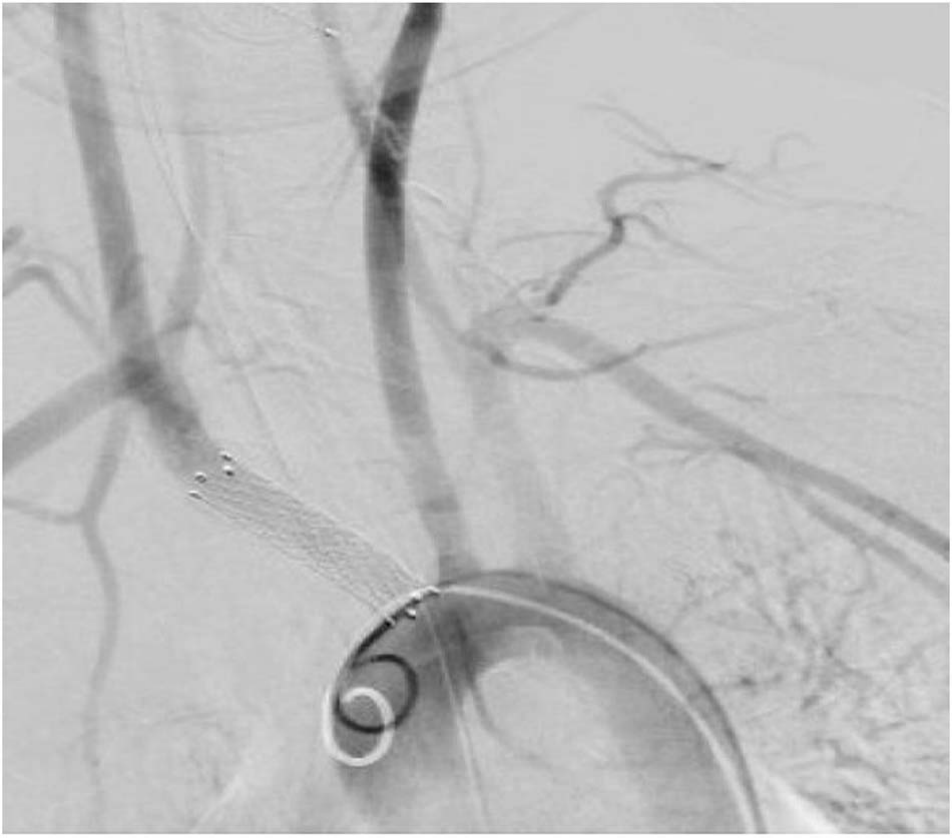
Angiogram post endovascular stenting.

Endovascular stent grafting of the IA aneurysm was performed through femoral artery access. Device selection was guided by key anatomical and clinical considerations: the pseudoaneurysm had proximal and distal landing zones measuring 7 and 8 mm in diameter and was located 5 mm from the origin of the IA, at a sufficient distance from the carotid and subclavian branches. A 10 × 40-mm Fluency Plus stent graft was chosen to match the vessel diameter, provide adequate coverage of the 9 mm intimal flap, and ensure a secure seal while maintaining patency of the carotid and subclavian arteries. The graft’s length allowed for proper anchoring in the available landing zones, and its flexibility facilitated navigation in the curved aortic arch. Final aortogram confirmed exclusion of the pseudoaneurysm with preserved carotid and subclavian flow (Fig. 4).

He underwent several maxillofacial and orthopedic interventions. After 35 days, he was transferred for rehabilitation. At the time of discharge, he was able to ambulate independently with minimal assistance.

## Discussion

IAI is a rare but life-threatening complication of blunt trauma, mostly associated with high-speed motor vehicle collisions [4]. The injury mechanisms involve compressive forces on the mediastinum, which displace the heart posteriorly and to the left, increasing tension on the aortic arch and thoracic outlet vessels [5].

Patients with IAI often sustain polytrauma and high mortality [6]. Prompt recognition and high clinical suspicion are crucial for successful management. CT angiography remains the diagnostic gold standard, providing clear vascular visualization [4]. Open surgical repair is the standard and most common approach. This often requires a median sternotomy extended to the right neck for exposure [7]. However, this method has high morbidity and mortality, especially in unstable trauma patients with multiple injuries [8, 9].

Recently, endovascular repair has emerged as a less invasive alternative. It offers reduced perioperative risks [1]. While the number of reported cases is still limited, available comparative data suggest a lower in-hospital mortality rate with endovascular techniques compared to open surgical repair. In published series, open repair mortality ranges from 20% to 25%, whereas endovascular management shows rates as low as 8%–10%. Additionally, neurological complications, such as perioperative stroke, appear less frequent in endovascular cohorts. These findings highlight a tangible advantage favoring endovascular intervention, particularly in carefully selected patients. These procedures are best performed in hybrid operating theaters with surgical teams on standby [10]. Only a limited number of IAIs have been reported. Table 1 summarizes reported cases of IAIs managed with endovascular repair [1, 5, 10–14].

**Table 1:** Literature review of blunt IA injury managed with endovascular repair.

Author	Year	No of cases	Gender age (years)	Mechanism of injury	IA injury	Concomitant injuries	Stent used	Follow-up/Neurological status
Miles *et al.* [11]	2003	1	Male 29	MVC	Transection of IA 1.5 cm from origin No extravasation Large left mediastinal hematoma	Dissection and thrombus of left proximal vertebral artery Right orbital fracture Multiple ribs fracture	12 × 30 mm Wallgraft endoprosthesis stent	No available data about long-term follow-up No neurological deficit on discharge
Huang *et al.* [5]	2008	1	Male 36	MVC	Pseudoaneurysm, 1.8 cm diameter involving IA to RSA and CCA	Orbital rim fracture Bilateral hemo-pneumothorax Right big toe fracture	Two overlapping Wallgraft endoprosthesis (10 × 50 mm and 12 × 30 mm, Boston Scientific Corporation, Galway, Ireland)	12 months No graft thrombosis No neurological deficit
Hu *et al.* [10]	2020	1 (1/10)	Male 25	Trauma (not specified)	Pseudoaneurysm Diameter of IA: 10 mm	Not specified	Kissing Viabahn stent, graft dimensions (CCA, RSA) 8 × 100, 10 × 100 mm	Immediate outcome: small type I endoleak 6 and 12 months: small residual Type I endoleak 24 months: stent in good position and no endoleak No neurological deficit
Jia *et al.* [1]	2020	3 (3/7)	Male 34	RTA	Intimal tear	Sternal fracture	Covered stent and balloon The diameter of the stent was measured by arteriography with ~ 10% oversize	15 months Doppler: no stenosis, occlusion, thrombosis No neurological deficit
			Male 43	Crush injury	Partial transection	Rib fracture and craniocerebral injury		12 months Doppler: no stenosis, occlusion, thrombosis No neurological deficit
			Female 27	RTA	Intimal tear	Rib and clavicular fracture		24 months Doppler: no stenosis, occlusion, thrombosis No neurological deficit
Pereira-Neves *et al.* [12]	2021	1	Male 49	MCC	29 mm pseudoaneurysm of the right IA and its bifurcations Enlarged mediastinum	Multiple fractures; Le fort Type II right facial, multiple ribs right clavicle and right patella	12 × 58 mm CBES (Lifestream Bard) followed by balloon dilatation 14 × 40 mm Kissing stent with two CBES of IA bifurcation (10 × 58 mm in the RSA and 7 × 58 mm in the right CCA	10 days after discharge Lost follow-up afterwards No neurological deficit
Kota *et al.* [13]	2021	1	Male 29	RTA	IA pseudoaneurysm and proximal right CCA dissection	Multiple fractures involving the skull, face, ribs, and extremities and grade 1 hepatic and grade 3 splenic blunt injuries	Parallel self-expanding covered stents (Fluency, Bard, Ariz) Extending from the IA ostium into the mid-right CCA and proximal RSA using dual femoral arterial percutaneous access	12 months: patent stent No neurological deficit
Dang *et al.* [14]	2022	1	Female 20	MVC	pseudoaneurysm of IA extending from the origin the bifurcation of RSA and CCA No extravasation Large mediastinal hematoma	Small bilateral pneumothorax Right mandibular fracture	10 × 39-mm Viabahn VBX stent	1 and 6 months: patent IA and CCA. No neurological deficit.

Abbreviations: CBES, covered balloon expandable stent; CCA, common carotid artery; IA, innominate artery; MCC, motorcycle crash; MVC, motor vehicle collision; RSA, right subclavian artery; RTA, road traffic accident.

In our case, the patient had a blunt IAI from a motorcycle accident. He initially presented with hemodynamic instability. CT imaging confirmed a proximal IAI, about 5 mm from its origin, with an intimal flap and contained contrast extravasation.

Because of high morbidity and mortality in IAIs, a multidisciplinary team was vital. Early coordination included trauma surgeons managing resuscitation and triage, radiologists promptly conducting and interpreting advanced imaging to localize the vascular injury. Vascular and cardiothoracic surgeons jointly participating in decision-making regarding repair options. The patient stabilized in the trauma ICU under intensive monitoring by critical care specialists. Imaging was repeated to confirm the injury’s location and extent. As the patient’s condition improved and the aortic arch remained uninvolved, vascular and cardiothoracic teams determined that endovascular stenting was feasible, with anesthesiology and interventional radiology support. The procedure was performed using a self-expanding stent in a hybrid theater, but in centers without a hybrid suite, close collaboration between interventional radiologists and surgeons in either an operating room or angiography suite is crucial. Intraoperative aortography confirmed the exclusion of the pseudoaneurysm and the patency of the carotid and subclavian arteries. To guide similar centers, we suggest a decision algorithm that emphasizes early multidisciplinary evaluation, recurrent imaging, and joint assessment of anatomic suitability and patient stability for selecting between endovascular stenting and open repair. The patient had no immediate or short-term complications during the first postoperative month.

## Conclusion

Blunt IAI is a rare and high-risk vascular trauma that requires prompt and accurate diagnosis, as well as appropriate management. This case report demonstrates that endovascular stent grafting can provide a viable, less invasive alternative to open surgical repair for blunt injuries to the IA.

## Data Availability

Data sharing does not apply to this article as no new data were created or analyzed in this study.
